# Data-independent acquisition-based quantitative proteomic analysis of m.3243A>G MELAS reveals novel potential pathogenesis and therapeutic targets

**DOI:** 10.1097/MD.0000000000030938

**Published:** 2022-10-14

**Authors:** Xueli Chang, Zhaoxu Yin, Wei Zhang, Jiaying Shi, Chuanqiang Pu, Qiang Shi, Juan Wang, Jing Zhang, Li Yan, Wenqu Yang, Junhong Guo

**Affiliations:** a Department of Neurology, First Hospital of Shanxi Medical University, Taiyuan, Shanxi, China; b Department of Neurology, Chinese People’s Liberation Army General Hospital, Beijing, China; c Department of Anesthesiology, Shanxi Bethune Hospital, Shanxi Academy of Medical Science, Taiyuan, Shanxi, China.

**Keywords:** DIA, encephalopathy, lactic acidosis and stroke like episodes (MELAS), mitochondrial myopathy, proteomics, skeletal muscle

## Abstract

The pathogenesis of mitochondrial myopathy, encephalopathy, lactic acidosis and stroke like episodes (MELAS) syndrome has not been fully elucidated. The m.3243A > G mutation which is responsible for 80% MELAS patients affects proteins with undetermined functions. Therefore, we performed quantitative proteomic analysis on skeletal muscle specimens from MELAS patients. We recruited 10 patients with definitive MELAS and 10 *age- and gender- matched* controls. *Proteomic analysis based on* nanospray liquid chromatography-mass spectrometry (LC-MS) was performed *using* data-independent acquisition (DIA) *method* and differentially expressed proteins were revealed by bioinformatics analysis. We identified 128 differential proteins between MELAS and controls, including 68 down-regulated proteins and 60 up-regulated proteins. The differential proteins involved in oxidative stress were identified, *including* heat shock protein beta-1 (HSPB1), alpha-crystallin B chain (CRYAB), heme oxygenase 1 (HMOX1), glucose-6-phosphate dehydrogenase (G6PD) and selenoprotein P. *Gene ontology and* kyoto encyclopedia of genes and genomes pathway analysis showed significant enrichment in phagosome, *ribosome and* peroxisome proliferator-activated receptors (PPAR) signaling pathway. The imbalance between oxidative stress and antioxidant defense, *the* activation of autophagosomes, and *the* abnormal metabolism of mitochondrial ribosome proteins (MRPs) might play an important role in m.3243A > G MELAS. The combination of proteomic and bioinformatics analysis could contribute *potential* molecular networks to the pathogenesis of MELAS in a comprehensive manner.

## 1. Introduction

Mitochondrial diseases are one of the most common congenital metabolic defects with a minimum prevalence of 1:5000.^[1,2]^ Mitochondrial myopathy, encephalopathy, lactic acidosis and stroke like episodes (MELAS) is one of the *most frequent* mitochondrial diseases affecting the central nervous system. MELAS *is characterized by systemic* clinical manifestations including stroke, recurrent headaches, epilepsy, hearing impairment, myopathy, dementia, ataxia and diabetes.^[3]^ The m.3243A > G mutation in the MT-TL1 gene^[4,5]^ accounts for almost 80% of the abnormalities in MELAS patients. The mutation disturbs mitochondrial protein synthesis and ultimately affect the assembly of Complexes I, III, and IV on oxidative respiration chains.^[6]^ *However*, the molecular *basis* underlying the pathogenesis of MELAS is still poorly understood. *Therefore*, it is *crucial* to unravel its pathogenesis at molecular level *using more effective diagnostic methods to* reduce complications in MELAS.

Mitochondrial proteome is co-encoded by mtDNA and nuclear genome. The mtDNA encodes 13 proteins *that are essential components of oxidative phosphorylation (OXPHOS*); while the nuclear genome encodes the remaining roughly 1500 proteins that are transferred to the mitochondria through complex import systems.^[7]^ The functions of mutated proteins in mitochondrial diseases remain undetermined, the discovery of which might shed light on its pathogenesis. Recently, the combination use of high-throughput omics techniques and complex bioinformatics analysis have brought new hope for revealing the pathogenesis and therapeutic targets of mitochondrial diseases. The quantitative proteomics and bioinformatics analysis could facilitate the understanding of the post-translational modifications.^[8]^ Compared to data-dependent-acquisition (DDA) method, data-independent acquisition (DIA) method *produces* more reliable results using a smaller size of samples. Accordingly, the DDA-based proteomics is gradually replaced by DIA-based proteomics which is increasingly applied to the study of various diseases^[9]^ as a new tool.

In this study, we performed nanospray liquid chromatography-mass spectrometry (LC-MS) based proteomic analysis in the DIA modes on skeletal muscle specimens from patients with MELAS to reveal the differentially expressed proteins and identify potential signaling pathways for a better understanding of the pathogenesis.

## 2. Materials and Methods

### 2.1. Characteristics of individuals and muscle biopsy

The diagnostic criteria of MELAS were as follows in this study: (1) **Category A** clinical findings of stroke-like episodes: headache with vomiting; seizure; hemiplegia; cortical blindness or hemianopsia; acute focal lesion observed via brain imaging. (2) **Category B**: evidence of mitochondrial dysfunction: high lactate levels (2 mmol/L or more) in plasma and/or cerebral spinal fluid or deficiency of mitochondrial-related enzyme activities; mitochondrial abnormalities in muscle biopsy; definitive gene mutation related to MELAS; (3) **Definitive MELAS**: 2 items of Category A and 2 items of Category B (4 items or more).^[10]^

In this study, all subjects underwent muscle biopsy for diagnostic purposes. Muscle biopsies were obtained from quadriceps femoris or biceps brachii. We selected the MELAS patients with point mutation for m.3243 A > G in genetic screening. We selected the age- and gender-matched subjects who were ultimately deemed to be free of neuromuscular diseases through muscle biopsies as controls.

### 2.2. Protein digestion

100 μg of protein from each sample was solubilized in a new Eppendorf tube containing 8 M urea to achieve a final volume of 100 μL. Then, 2 μL of 0.5 M TCEP was added to the sample for incubation for 1 hour at 37°C, along with 4 μL of 1 M iodoacetamide added and incubated in the dark at room temperature for another 40 minutes. Subsequently, -20°C pre-chilled acetone were added to the sample at the ratio of 5:1, precipitated at -20°C overnight, and centrifuged for 20 minute at 12,000 G. Precipitates were washed by 1 mL pre-chilled 90% acetone aqueous solution and centrifuged for twice. Samples were re-dissolved in 100 μL 100 mM TEAB and digested by Sequence grade modified trypsin (Promega, Madison, WI) containing 1:50 enzyme: protein (weight: weight) at 37°C overnight. The peptide mixture was desalted by C18 ZipTip followed by quantification with Pierce™ Quantitative Colorimetric Peptide Assay (23275). SpeedVac was used for lyophilization.

### 2.3. Establishment of spectrum database

#### 2.3.1. High PH reverse phase separation.

The mixed peptides were re-dissolved in buffer A (buffer A: 20 mM ammonium formate in water, pH 10.0, adjusted with ammonium hydroxide) and then loaded onto a reverse phase column (XBridge C18 column, 4.6 mm × 250 mm, 5 μm, Waters Corporation, MA) using Ultimate 3000 HPLC system (Thermo Fisher scientific, MA). High pH separation was achieved in 40 minute with a linear gradient starting from 5% B to 45% B (B: 20 mM ammonium formate in 80% ACN, pH 10.0, adjusted with ammonium hydroxide). The column was re-equilibrated at 30°C for 15 minute (flow rate: 1 mL/min). A total of 10 fractions were obtained and dried by vacuum concentrator for future use.

#### 2.3.2. Nano-hplc-MS/MS analysis.

The peptides were re-dissolved in solvent A (A: 0.1% formic acid in water) and on-line nanospray LC-MS/MS was performed for analysis on an Orbitrap Lumos coupled to EASY-nLC 1200 system (Thermo Fisher Scientific, MA). 4 μL peptide sample was loaded onto analytical column (Acclaim PepMap C18, 75 μm × 25 cm) and eluted with 120-minute gradient from 4% to 32% B (B: 0.1% formic acid in ACN). We set the column flow rate at 300 nL/min and the capillary voltage at 2 kV. We *ran* the mass spectrometer under DDA mode with MS and MS/MS mode automatically switched. The parameters were as follows: MS: scan range (m/z) = 350 to 1500; resolution = 60,000; AGC target = 4e5; maximum injection time = 50 ms; dynamic exclusion = 30 second; HCD-MS/MS: resolution = 15,000; AGC target = 5e4; maximum injection time = 38 ms; collision energy = 32.

### 2.4. DIA-MS data processing

#### 2.4.1. Nano-hplc-MS/MS analysis.

The samples were re-dissolved in 30 μL solvent A (A: 0.1% formic acid in water). 9μL of each was taken, added with 1μL 10 × iRT peptide, mixed, seperated with nano-LC, and finally analyzed by on-line nanospray LC-MS/MS on an Orbitrap Lumos coupled to EASY-nLC 1200 system (Thermo Fisher Scientific, MA). 4 μL peptide sample was loaded onto analytical column (Acclaim PepMap C18, 75 μm × 25 cm) and eluted with 120-minute gradient from 4% to 32% B (B: 0.1% formic acid in ACN). We set the column flow rate at 300 nL/min and the capillary voltage at 2 kV. We *ran* the mass spectrometer under DIA mode with MS and MS/MS mode automatically switched. The parameters were as follows: MS: scan range (m/z) = 350 to 1350; resolution = 120,000; AGC target = 4e5; maximum injection time = 50 ms; HCD-MS/MS: resolution = 30,000; AGC target = 1e6; collision energy = 32; 60 variable Isolation windows was set with each window overlapped 1 m/z.

#### 2.4.2. Data analysis.

We used Spectronaut X (Biognosys AG, Switzerland) with default settings to process and analyze the raw data of DIA. Retention time prediction type was set to dynamic iRT and automatically determine the ideal extraction window. The criteria for protein identification were as follows: Q value (FDR) cutoff on precursor and protein level were both applied to 1%. The Decoy database was generated using a mutated strategy similar to scrambling a random number of amino acids (min = 2, max = length/2). All selected precursors that meet the screening conditions were used for quantification. All interfering fragment ions were excluded by MS2 interference except for the 3 least interfering ones. The major group quantities were computed using the average top 3 filtered peptides (> the 1% Qvalue cutoff). Differential expressed proteins were filtered if their *P* value <.05 and fold change > 1.3 using Welch’s ANOVA Test.

### 2.5. Bioinformatics analysis

The partial least-squares-discriminant analysis, a classic PLS regression for solving discrimination and classification problems, was performed by mixOmics package (https://CRAN.R-project.org/package=mixOmics). Hierarchical clustering analysis *was performed using* the pheatmap package (https://CRAN.R-project.org/ package = pheatmap). The volcano plot *was generated by ggplot2 package for visualization.* Functional annotation was performed by Blast2GO version 5 and gene ontology analysis was performed using GOATOOLS.^[11,12]^ Kyoto Encyclopedia of Genes and Genomes pathway enrichment analysis was performed by KOBAS (http://kobas.cbi.pku.edu.cn/).^[13]^ *The P <.05 was considered to be statistically significant. Meanwhile, the adjusted p values were obtained to decrease the false positive rate using the Hochberg false discovery rate and the Benjamini method.* The protein-protein interaction (PPI) network was constructed using STRING v10 (www.string-db.org),^[14]^ *a database containing information about experimental and predicted interactions of proteins. The interactions include direct (physical) interactions and indirect (functional) interactions. A combined confidence score of ≥ 0.4 was used as the cuff-off value. In the PPI network, nodes represents proteins and edges represent the relationships among them.*

## 3. Results

### 3.1. Clinical manifestations

10 patients (5 males, 5 females) diagnosed with definitive MELAS according to the diagnostic criteria were recruited from specialist clinics in our hospital. The median age of the patients was 38.5 years (range 11–52). Clinical features of MELAS patients *were* shown in Table 1. The mutation heteroplasmy ranged from 56% to 89% in muscle tissue. The age- and gender-matched controls consisted of 10 healthy subjects (5 males, 5 females) without neuromuscular diseases evaluated by 2 specialists. The median age was 39.5 years (range 12–53).

**Table 1:** Characteristics of the MELAS (mt 3243A > G) patients.

No	Sex	Age	Onset of age	Clinical symptoms	Lactate level in plasma at rest	% muscle heteroplasmy	*Biopsied muscle*	Muscle biopsy	Head MRI
1	F	11	10	seizures, headache, myopathy	2.2 mmol/L	75	*biceps*	G(+), S(-)	left temporoparietal lobes
2	M	47	42	hemiplegia, hearing loss, impaired vision, diabetes, ataxia	2.5 mmol/L	70	*quadriceps*	G(-), S(+)	right occipital lobe and left temporoparietal lobes
3	F	26	23	seizures, headache, diabetes, short stature, hearing loss	4.5 mmol/L	83	*quadriceps*	G(+), S(+)	right temporal lobe
4	F	45	40	diabetes, impaired vision, seizures, short stature	1.9 mmol/L	65	*quadriceps*	G(+), S(+)	right occipital and temporal lobes
5	M	52	49	blepharoptosis, seizures, hearing loss, hemiplegia, short stature	1.8 mmol/L	56	*biceps*	S(+), C(+)	bilateral temporal lobes
6	M	49	45	seizures, headache, short stature, diabetes, impaired vision	3.1 mmol/L	70	*biceps*	G(-), S(+)	left temporal and right occipital lobe
7	M	31	30	headache, myopathy, hemiplegia, impaired vision, diabetes	3.4 mmol/L	79	*quadriceps*	G(+), S(+)	left temporal and occipital lobes
8	F	50	48	seizures, hemiplegia, diabetes, short stature	4.3 mmol/L	80	*biceps*	G(+), S(-)	left temporal lobe, right temporoparietal lobes
9	F	32	30	headache, myopathy, seizures, ataxia, impaired glucose tolerance	3.3 mmol/L	80	*biceps*	G(+), S(-)	left temporoparietal lobes
10	M	29	25	blepharoptosis, myopathy, ataxia, seizures, headache, hearing loss	2.1 mmol/L	89	*quadriceps*	G(+), S(+)	bilateral temporoparietal lobes

G(+): ragged-red fiber (RRF) in MGT staining; S(+): strongly SDH-reactive blood vessels (SSV) or ragged-blue fibers (RBFs) in SDH staining; C(+): COX deficient fibers in COX staining.

MELAS = mitochondrial myopathy, encephalopathy, lactic acidosis and stroke like episodes, MRI = magnetic resonance imaging.

### 3.2. Identification of the *differentially expressed proteins* between MELAS and controls

**Figure 1.**
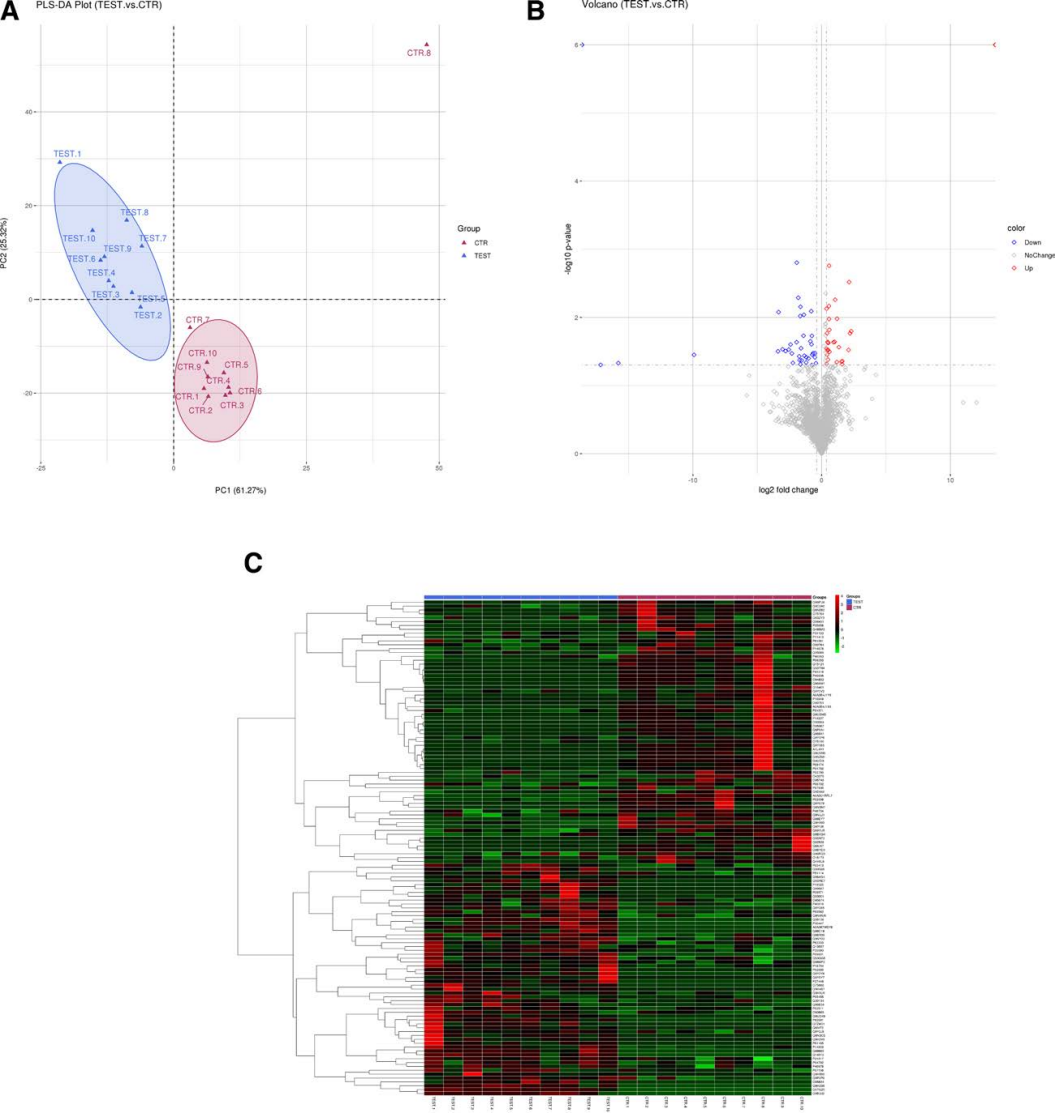
**The PLS-DA model, the volcano plots and heat map of differential proteins of the MELAS and control groups**. (A) *The PLS-DA model of differential proteins.* All observed MS peaks *were* included in the model; Clear separation of the *2* groups *was* observed. (B) *The volcano plots of the differential proteins. Dots* in red represent up-regulated *proteins*, and dots in green represent down-regulated proteins, *and dots in gray represent proteins without any aignificant difference.* (C) *Heatmap of the differential proteins.* TEST.1 − TEST.10 represent replicates in the MELAS group and CTR.1 − CTR.10 represent replicates, in the control group. The up-regulated proteins are *presented* in red, and the down-regulated proteins are presented in green. TEST: MELAS patients; CTR = control, MELAS = Mitochondrial myopathy, encephalopathy, lactic acidosis and stroke like episodes, MS = mass spectrometry, PLS-DA = partial least-squares-discriminant analysis.

The partial least-squares-discriminant analysis model showed significant difference, indicating a good *classification* between the 2 groups (Fig. 1A). The Volcano plots revealed 128 differential *expressed* proteins (Supplementary 1, http://links.lww.com/MD/H459) between MELAS and controls, including 68 down-regulated proteins and 60 up-regulated proteins (Fig. 1B). Heat map analysis exhibited levels of differential *expressed* proteins, providing information on the underlying metabolic *disturbance* caused by m.3243A > G (Fig. 1C). These results suggested that despite of the donor variation, the patterns of differential proteins expression varied between these 2 groups.

The expression of protein involved in glycogen synthesis (Glycogenin-1) was significantly increased in MELAS patients. Interestingly, the expression of HADHA which is related to oxidation of fatty acids was up-regulated while choline dehydrogenase was down-regulated compared to controls. The PTCD3 (related to mitochondrial translation) and MICU2 (a mitochondrial inner membrane protein) were not expressed at all in MELAS patients. Finally, we analyzed the expression differences in proteins involved in oxidative stress: G6PD expression in the skeletal muscle was significantly down-regulated in MELAS patients. Selenoprotein P was not expressed at all in MELAS patients. On the contrary, the expression of HSPB1, CRYAB and S100A9 were significantly up-regulated in MELAS patients. The HMOX1 was not expressed at all in controls (Supplementary 2, http://links.lww.com/MD/H460).

### 3.3. Functional enrichment analysis

**Figure 2.**
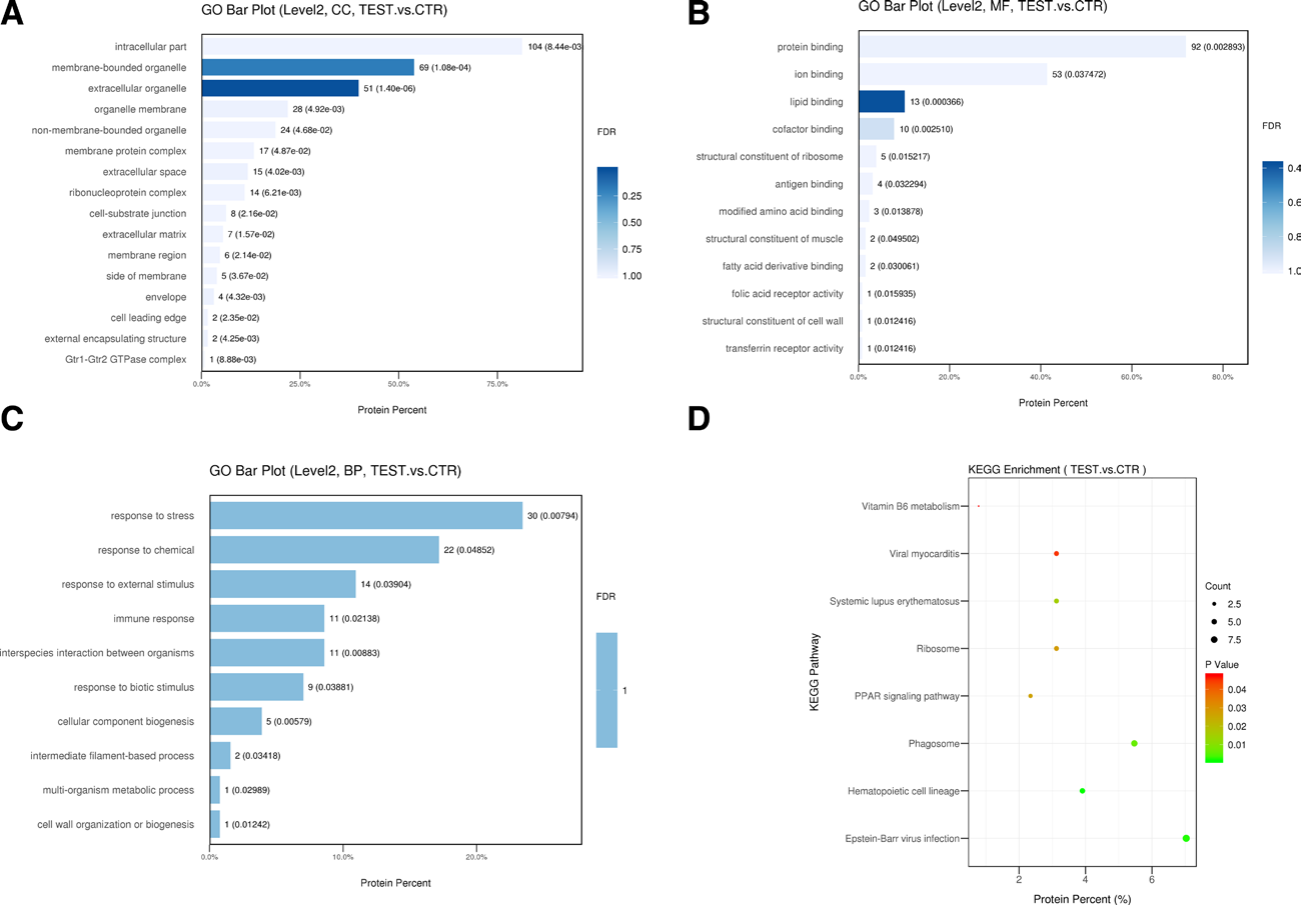
GO analysis of the differential proteins and KEGG pathway enrichment analysis in MELAS and control. (A) Cellular components, (B) molecular functions, (C) biological process, (D) KEGG pathway enrichment analysis. *P* <.05 *was considered statistically significant*. TEST: MELAS patients; CTR = control, GO = gene ontology, KEGG = Kyoto encyclopedia of genes and genomes, MELAS = Mitochondrial myopathy, encephalopathy, lactic acidosis and stroke like episodes.

*In the gene ontology enrichment analysis, the categories include* cellular component, molecular function and biological process o*f differentially expressed proteins*. For the cellular component category, the differential proteins were mostly enriched in intracellular part, membrane-bounded organelle and extracellular organelle (Fig. 2A). For the molecular function category, the differential proteins were predominantly involved in protein binding, iron binding and lipid binding (Fig. 2B). For the biological process category, the differential proteins were mainly associated with the response to stress, response to chemical, response to external stimulus, immune response and interspecies interaction between organisms (Fig. 2C). Kyoto encyclopedia of genes and genomes pathway enrichment analysis indicated significant enrichment in hematopoietic cell lineage, Epstein-Barr virus infection, phagosome, systemic lupus erythematosus, peroxisome proliferator-activated receptors (PPAR) signaling pathway, ribosome, viral myocarditis and vitamin B6 metabolism (Fig. 2D).

### 3.4. PPI network *construction*

**Figure 3.**
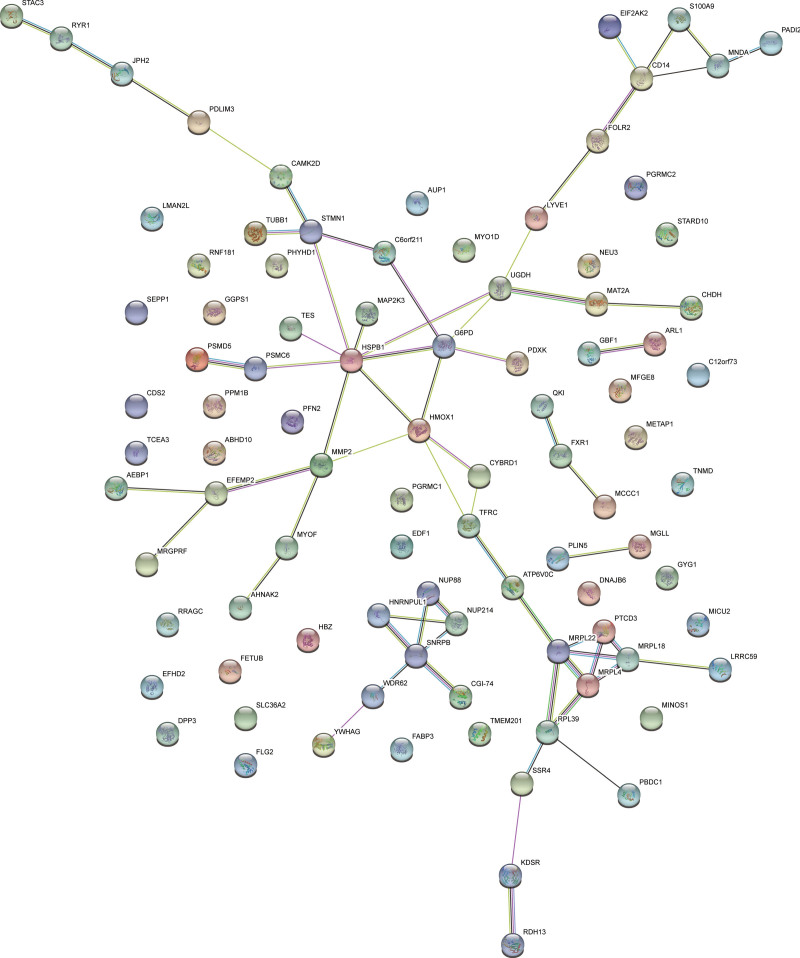
PPI network of differential proteins. The *nodes* represent proteins *and edges represent interactions among the nodes*. Results inside the *nodes* represent protein structure. PPI = protein-protein interaction.

PPI network was constructed using the STRING database to illustrate the interactions *among the* differential proteins (Fig. 3). A total of 128 proteins were used as search inputs, 93 of which were matched in the database, including 43 up-regulated proteins and 50 down-regulated proteins. 11 *potential* key proteins were *identified* in the PPI network, including HSPB1, G6PD, HMOX1, SNRPB, MRPL22, STMN1, MMP2, UGDH, CD14, MRPL4, and MRPL18. *Notably, HSPB1 connecting 8 nodes was considered as the most significant.* HSPB1, HMOX1, CD14, MRPL22 and MRPL18 in the module were up-regulated and G6PD, SNRPB, STMN1, MMP2, UGDH and MRPL4 were down-regulated.

## 4. Discussion

*To our best knowledge*, this is the first proteomes analysis of skeletal muscle collected from m.3243A > G MELAS patients compared with controls using the method of the DIA *in the literatures*. We found 128 differentially expressed proteins, including 68 down-regulated proteins and 60 up-regulated proteins. The proteins involved in oxidative stress *were* analyzed, indicating a highly significant up-regulation in HSPB1, CRYAB, and HMOX1 and a down-regulation in G6PD and selenoprotein P. The most significantly differentially expressed proteins were HSPB1 and CRYAB, both of which are small heat shock proteins (sHsp). They could combine misfolded proteins to prevent denaturation, inhibit cellular apoptosis and serve as molecular chaperones to regulate the intracellular redox state.^[15,16]^ In mutant HSPB1 expressing motor axons, decreased mitochondrial Complex I activity and increased mitochondrial vulnerability were observed, leading to increased superoxide release and decreased mitochondrial glutathione levels.^[17]^ CRYAB could protect cells from hypoxia, maintain mitochondrial integrity^[18,19]^ and act in vascular biology.^[20]^ CRYAB *is* crucial for endothelial cell survival in hypoxia.^[19,21,22]^ sHsps could attenuate mitochondrial dysfunctions, block oxidative stress and minimize neuronal apoptosis. Therefore, sHsps *are* regarded as promising protectants in some neurodegenerative diseases.^[23]^ G6PD, the first rate-limiting enzyme in the pentose phosphate pathway, *is* indispensable to maintain oxidation-reduction equilibrium in cells.^[24]^ Several lines of evidence suggested that sHsp protects against oxidative stress *by* increasing G6PD activity and maintaining optimal cellular detoxifying machinery.^[16]^ *Interestingly*, our results showed that G6PD was down-regulated, different from results obtained in another study.^[25]^ *As for the contradictory result with respect to G6PD, we speculated that there were several potential factors: firstly, these MELAS patients had different severity and duration in the 2 studies, while the muscle tissue obtained only provided limited information regarding a certain stage in the course of MELAS; secondly, the pathogenesis of MELAS was extremely complicated; thirdly, sHsps might protect against anti-oxidative stress through other pathways rather than regulating G6PD-dependent ability. In addition, we found that HMOX1, not expressed in human skeletal muscle* (https://www.proteinatlas.org/*), was highly up-regulated in MELAS while it was not expressed in controls. This was consistent with another study which evidenced the upregulation of HMOX1 in induced pluripotent stem cell of a MELAS patient, confirming higher oxidative stress in MELAS.*^[26]^ HMOX1 over-expression *has also been* demonstrated to be associated with intracellular oxidative stress, mitochondrial dysfunction and mitophagy.^[27]^ Increased levels of HMOX1 have been observed in many neurodegenerative diseases including Alzheimer’s *disease*, Parkinson’s disease, etc.^[27]^ Thus, we speculated that these oxidative-related proteins might be associated with the *progression* and severity of clinical symptoms of MELAS.

*In functional enrichment analysis of our study*, pathways in phagosome, PPAR signaling, ribosome and vitamin B6 metabolism showed significant enrichment. Previous studies have found accumulation of autophagosomes in MELAS fibroblasts.^[28]^ *It is speculated that* the enrichment in autophagosome might be an adaptive response to intracellular OXPHOS and mitochondrial deficiency. As compensatory response, the increased autophagosome could clear damaged mitochondria to avoid further cellular dysfunction.^[29,30]^ On the contrary, the mitochondrial dysfunction blocks both autophagy induction and autophagic flux.^[31]^ Therefore, it is still controversial whether autophagy produces protective or harmful effects. Defects in phagosome are related to a variety of cellular metabolic process, including abnormal lipid metabolism, oxidative stress injury and mitochondrial dysfunction,^[32–34]^ whereas autophagosome accumulation could impair cell bioenergetics and also induce cell death.^[35]^ We proposed that the response to autophagosome varies widely, depending on the severity of mitochondrial deficiency. The phagosome pathway may be one of the essential molecular networks involved in the pathogenesis of MELAS.

PPAR signaling pathway is a crucial regulator for β oxidation of fatty acid^[36,37]^ and transcription of nuclear-encoded mitochondrial genes. Bezafibrate, as a pan-PPAR activator, could up-regulate the expression of Peroxisome proliferator-activated receptor gamma coactivator 1-alpha, promote mitochondrial biogenesis and improve mitochondrial deficiency of mitochondrial diseases.^[38]^ *Therefore*, the PPAR signaling pathway might be a potential disease-modifying therapeutic target.

The mitochondrial ribosome proteins (MRPs) are synthesized in cytoplasm, introduced into mitochondria for assembly and responsible for the translation of 13 mitochondrial mRNAs.^[39]^ In our study, MRPL22 and MRPL18 were up-regulated, while MRPL4 and PTCD3 were down-regulated. MRPL18 facilitates ribosome assembly to participate in selected mRNAs translation and stress regulation, contributing to a strong cytoplasmic stress response under stress conditions.^[40]^ Therefore, MRPL18 is one of a key regulator *of* cytoplasmic stress response. PTCD3, also known as MRPS39, is one of the mitochondrial ribosomal supernumerary proteins unique to mammals.^[41]^ A study showed that mitochondrial protein synthesis was seriously disturbed in PTCD3 knockdown cells, resulting in OXPHOS deficiency with no effect on RNA metabolism.^[42]^ PTCD3 mutations led to impaired translation of mtDNA-encoded proteins, resulting in combined defects of Complex I and IV, and decreased ATP production.^[43]^ Although MRPL4 has not been reported in the mitochondrial diseases, *it* has attracted increasing attention from researchers because it was reported to be *a* downstream *molecular* of hypoxia-inducible factor-1α.^[44]^ In summary, it’s tempting to speculate that MRPs might play a key role in the occurrence, *progression* and prognosis of MELAS.

However, this study also had certain limitations: first of all, *the sample size was relatively small which may* not accurately *represent* all MELAS patients. Secondly, despite an effort to minimize the individual differences, the skeletal muscle could only provide certain information about a specific stage in MELAS *progression*. Thirdly, the DIA protein library was established *based on* the DDA atlas database and might lead to a loss of some peptides *in* analysis. Therefore, larger sample size and more quantitative studies are needed to verify our results.

## 5. Conclusion

Our results *revealed* a large number of differentially *expressed* proteins in skeletal muscles of *m.3243A > G* MELAS patients *compared to controls*. In addition, the imbalance between oxidative stress and antioxidant defense, *the* activation of autophagosomes and *the* abnormal metabolism of MRPs might play a critical role in m.3243A > G MELAS patients. Further investigation of these proteins could contribute novel insights to the pathogenesis of MELAS in a comprehensive manner.

## Authors’ contributions

Junhong Guo raised and designed this subject. Xueli Chang and Zhaoxu Yin collected and extracted data, and drafted the article. Wei Zhang, Jiaying Shi and Li Yan analyzed the data. Chuanqiang Pu and Qiang Shi provided some muscle tissue samples. Juan Wang, Jing Zhang and Wenqu Yang interpreted the results and revised the manuscript.

**Conceptualization:** Chuanqiang Pu, Junhong Guo.

**Data curation:** Wei Zhang, Li Yan.

**Formal analysis:** Wei Zhang, Jiaying Shi, Li Yan.

**Funding acquisition:** Junhong Guo.

**Resources:** Chuanqiang Pu, Qiang Shi.

**Supervision:** Juan Wang, Jing Zhang.

**Validation:** Wenqu Yang.

**Writing – original draft:** Xueli Chang.

**Writing – review & editing:** Zhaoxu Yin, Jing Zhang, Wenqu Yang.

## Acknowledgements

We thank Wu Cuimei and Wang Rui for performing the muscle biopsies. We thank all participants and their families who have performed the muscle biopsy. We are grateful to the Precision Gene for performing the Sanger and NGS sequencing. We also thank the omicsolution company in Shanghai for providing technical support.

## Supplementary Material


