# Supplementary material for: Data-independent acquisition-based quantitative proteomic analysis of m.3243A>G MELAS reveals novel potential pathogenesis and therapeutic targets
Source: Medicine (Baltimore). 2022 Oct 14;101(41):e30938. doi: 10.1097/MD.0000000000030938 (PMC9575705; doi:10.1097/MD.0000000000030938)
Supplement: Supplementary file 1 [file medi-101-e30938-s001.pdf]

Supplementary 1 The differential proteins between MELAS and controls

Protein ID	Protein	Protein descriptions	Genes	Fold Change Ratio	P Value
P02511	CRYAB	Alpha-crystallin B chain	CRYAB	1.376931	0.0228972
P04792	HSPB1	Heat shock protein beta-1	HSPB1	1.489312	0.0105384
P21817	RYR1	Ryanodine receptor 1	RYR1	1.343464	0.03052
P46976	GLYG	Glycogenin-1	GYG1	1.300340247	0.007447
P61981	1433G	14-3-3 protein gamma	YWHAG	0.736673	0.047067
P62333	PRS10	26S proteasome regulatory subunit 10B	PSMC6	1.427848	0.023717
Q13557	KCC2D	Calcium/calmodulin-dependent protein kinase type II subunit delta	CAMK2D	1.304512	0.028763
Q9Y2J8	PADI2	Protein-arginine deiminase type-2	PADI2	1.402772	0.02363
P05413	FABPH	Fatty acid-binding protein, heart	FABP3	1.482315	0.0068
P46734	MP2K3	Dual specificity mitogen-activated protein kinase kinase 3	MAP2K3	0.695895	0.038529
Q53GG5	PDLI3	PDZ and LIM domain protein 3	PDLIM3	1.335911	0.017075
P14678	RSMB	Small nuclear ribonucleoprotein-associated proteins B	SNRPB	0.315839	0.009555
P31153	METK2	S-adenosylmethionine synthase isoform type-2	MAT2A	0.603279	0.035273
Q96MF2	STAC3	SH3 and cysteine-rich domain-containing protein 3	STAC3	1.336838	0.035282
Q9H993	ARMT1	Protein-glutamate O-methyltransferase	ARMT1	0.384182	0.009151
O00764	PDXK	Pyridoxal kinase	PDXK	0.377275	0.01859
O60869	EDF1	Endothelial differentiation-related factor 1	EDF1	1.404992	0.041596
P62891	RL39	60S ribosomal protein L39	RPL39	-	0.000001
Q5TGZ0	MIC10	MICOS complex subunit MIC10	MINOS1	-	0.000001
Q9C0A0	CNTP4	Contactin-associated protein-like 4	CNTNAP4	0.591648	0.018664
Q9Y5Y7	LYVE1	Lymphatic vessel endothelial hyaluronic acid receptor 1	LYVE1	-	0.000001
P35080	PROF2	Profilin-2	PFN2	1.493197	0.001751
Q9UQ49	NEUR3	Sialidase-3	NEU3	-	0.000001

Continued

Protein ID	Protein	Protein descriptions	Genes	Fold Change Ratio	P Value
P05455	LA	Lupus La protein	SSB	4.2591	0.03001
Q9BR39	JPH2	Junctophilin-2	JPH2	1.518271	0.015224
Q9UGM5	FETUB	Fetuin-B	FETUB	$6.79*10^{-6}$	0.049733
Q9Y5P6	GMPPB	Mannose-1-phosphate guanyltransferase beta	GMPPB	0.433565	0.04582
O94832	MYO1D	Unconventional myosin-Id	MYO1D	0	0.000001
P18754	RCC1	Regulator of chromosome condensation	RCC1	2.922386	0.044566
P06702	S10A9	Protein S100-A9	S100A9	0.578733	0.024818
P07108	ACBP	Acyl-CoA-binding protein	DBI	1.311881	0.04811
Q69YU5	CL073	Uncharacterized protein C12orf73	C12orf73	0.571078	0.008103
Q9Y383	LC7L2	Putative RNA-binding protein Luc7-like 2	LUC7L2	0	0.000001
Q9Y679	AUP1	Ancient ubiquitous protein 1	AUP1	0.0000176	0.046796
O15173	PGRC2	Membrane-associated progesterone receptor component 2	PGRMC2	0.697133947	0.033448
Q15121	PEA15	Astrocytic phosphoprotein PEA-15	PEA15	0.122220688	0.029355
Q8IUX7	AEBP1	Adipocyte enhancer-binding protein 1	AEBP1	0.095284812	0.031482
Q96PU8	QKI	Protein quaking	QKI	0.169919442	0.029682
A0A087WSY6	KVD15	Immunoglobulin kappa variable 3D-15	IGKV3D-15	-	0.000001
P08174	DAF	Complement decay-accelerating factor	CD55	0	0.000001
P14207	FOLR2	Folate receptor beta	FOLR2	0	0.000001
Q9BUJ2	HNRL1	Heterogeneous nuclear ribonucleoprotein U-like protein 1	HNRNPUL1	-	0.000001
Q9H0V9	LMA2L	VIP36-like protein	LMAN2L	-	0.000001
P53582	MAP11	Methionine aminopeptidase 1	METAP1	2.070641125	0.005496
P61165	TM258	Transmembrane protein 258	TMEM258	-	0.000001
Q9H0U6	RM18	39S ribosomal protein L18, mitochondrial	MRPL18	3.018337213	0.048635

Continued

Protein ID	Protein	Protein descriptions	Genes	Fold Change Ratio	P Value
Q99685	MGLL	Monoglyceride lipase	MGLL	-	0.000001
Q9NY33	DPP3	Dipeptidyl peptidase 3	DPP3	1.524184159	0.031907
P09601	HMOX1	Heme oxygenase 1	HMOX1	-	0.000001
P51114	FXR1	Fragile X mental retardation syndrome-related protein 1	FXR1	1.421862413	0.030046
Q08431	MFGM	Lactadherin	MFGE8	0.2126775	0.046572
A1L4H1	SRCRL	Soluble scavenger receptor cysteine-rich domain-containing protein	SSC5D	0	0.000001
P14923	PLAK	Junction plakoglobin	JUP	4.394571016	0.003026
Q5SNT2	TM201	Transmembrane protein 201	TMEM201	0	0.000001
Q9NUJ1	ABHDA	Mycophenolic acid acyl-glucuronide esterase, mitochondrial	ABHD10	0.630739117	0.033467
P49908	SEPP1	Selenoprotein P	SELENOP	0	0.000001
Q16401	PSMD5	26S proteasome non-ATPase regulatory subunit 5	PSMD5	0	0.000001
P40616	ARL1	ADP-ribosylation factor-like protein 1	ARL1	1.892469755	0.023101
Q9Y2V2	CHSP1	Calcium-regulated heat-stable protein 1	CARHSP1	0.393632531	0.036505
Q96RQ3	MCCA	Methylcrotonoyl-CoA carboxylase subunit alpha, mitochondrial	MCCC1	0.592645495	0.049883
Q9Y365	STA10	START domain-containing protein 10	STARD10	-	0.000001
O95834	EMAL2	Echinoderm microtubule-associated protein-like 2	EML2	-	0.000001
P19525	E2AK2	Interferon-induced, double-stranded RNA-activated protein kinase	EIF2AK2	-	0.000001
Q9H2S6	TNMD	Tenomodulin	TNMD	-	0.000001
Q9NZM1	MYOF	Myoferlin	MYOF	0	0.000001
Q9P0P0	RN181	E3 ubiquitin-protein ligase RNF181	RNF181	2.287157998	0.010449
P67936	TPM4	Tropomyosin alpha-4 chain	TPM4	0.31879477	0.04882
Q96C19	EFHD2	EF-hand domain-containing protein D2	EFHD2	-	0.000001
Q05086	UBE3A	Ubiquitin-protein ligase E3A	UBE3A	0.299428184	0.037286

Continued

Protein ID	Protein	Protein descriptions	Genes	Fold Change Ratio	P Value
Q63ZY3	KANK2	KN motif and ankyrin repeat domain-containing protein 2	KANK2	0.494083167	0.039917
Q7Z4G1	COMD6	COMM domain-containing protein 6	COMMD6	-	0.000001
P46063	RECQ1	ATP-dependent DNA helicase Q1	RECQL	0.543700333	0.022409
P27449	VATL	V-type proton ATPase 16 kDa proteolipid subunit	ATP6V0C	-	0.000001
Q9UGI8	TES	Testin	TES	0	0.000001
Q00G26	PLIN5	Perilipin-5	PLIN5	4.607484125	0.017134
P01782	HV309	Immunoglobulin heavy variable 3-9	IGHV3-9	0.20138509	0.033812
P35658	NU214	Nuclear pore complex protein Nup214	NUP214	0	0.000001
Q96AG4	LRC59	Leucine-rich repeat-containing protein 59	LRRC59	-	0.000001
A0A0B4J1X8	HV343	Immunoglobulin heavy variable 3-43	IGHV3-43	0	0.000001
O75190	DNJB6	DnaJ homolog subfamily B member 6	DNAJB6	0.339742764	0.042703
P08571	CD14	Monocyte differentiation antigen CD14	CD14	-	0.000001
Q06136	KDSR	3-ketodihydrosphingosine reductase	KDSR	-	0.000001
Q53TN4	CYBR1	Cytochrome b reductase 1	CYBRD1	0	0.000001
Q5SRE7	PHYD1	Phytanoyl-CoA dioxygenase domain-containing protein 1	PHYHD1	-	0.000001
P16949	STMN1	Stathmin	STMN1	0	0.000001
Q8NE62	CHDH	Choline dehydrogenase, mitochondrial	CHDH	0.329519729	0.028366
P08253	MMP2	72 kDa type IV collagenase	MMP2	0.098237471	0.008352
P51571	SSRD	Translocon-associated protein subunit delta	SSR4	0.3204472	0.006978
Q03001	DYST	Dystonin	DST	3.047720346	0.043784
Q99567	NUP88	Nuclear pore complex protein Nup88	NUP88	-	0.000001
Q92538	GBF1	Golgi-specific brefeldin A-resistance guanine nucleotide exchange factor 1	GBF1	0	0.000001
Q30134	2B18	HLA class II histocompatibility antigen, DRB1-8 beta chain	HLA-DRB1	2.525769328	0.027302

Continued

Protein ID	Protein	Protein descriptions	Genes	Fold Change Ratio	P Value
Q6P0A1	F180B	Protein FAM180B	FAM180B	0	0.000001
O00264	PGRC1	Membrane-associated progesterone receptor component 1	PGRMC1	0	0.000001
Q5D862	FILA2	Filaggrin-2	FLG2	0.437900338	0.038503
Q4V9L6	TM119	Transmembrane protein 119	TMEM119	0.259298101	0.02311
Q8IVF2	AHNK2	Protein AHNAK2	AHNAK2	-	0.000001
O75688	PPM1B	Protein phosphatase 1B	PPM1B	-	0.000001
Q9HB90	RRAGC	Ras-related GTP-binding protein C	RRAGC	-	0.000001
P11413	G6PD	Glucose-6-phosphate 1-dehydrogenase	G6PD	0.143321113	0.031176
Q96B54	ZN428	Zinc finger protein 428	ZNF428	4.911935653	0.015957
Q9NSC5	HOME3	Homer protein homolog 3	HOMER3	-	0.000001
P41218	MNDA	Myeloid cell nuclear differentiation antigen	MNDA	0	0.000001
O95674	CDS2	Phosphatidate cytidylyltransferase 2	CDS2	2.267002373	0.045102
Q9BVG4	PBDC1	Protein PBDC1	PBDC1	0.284254844	0.005141
O95967	FBLN4	EGF-containing fibulin-like extracellular matrix protein 2	EFEMP2	0	0.000001
Q14515	SPRL1	SPARC-like protein 1	SPARCL1	-	0.000001
P30447	1A23	HLA class I histocompatibility antigen, A-23 alpha chain	HLA-A	-	0.000001
A0A0U1RRL7	MMPOS	Protein MMP24OS	MMP24OS	0	0.000001
P02786	TFR1	Transferrin receptor protein 1	TFRC	0.305159292	0.04236
Q9NWU5	RM22	39S ribosomal protein L22, mitochondrial	MRPL22	2.019234356	0.022818
Q9UNN5	FAF1	FAS-associated factor 1	FAF1	0	0.000001
O43379	WDR62	WD repeat-containing protein 62	WDR62	0.187461885	0.025003
Q9H4B7	TBB1	Tubulin beta-1 chain	TUBB1	-	0.000001
Q495M3	S36A2	Proton-coupled amino acid transporter 2	SLC36A2	0	0.000001

Continued

Protein ID	Protein	Protein descriptions	Genes	Fold Change Ratio	P Value
O75764	TCEA3	Transcription elongation factor A protein 3	TCEA3	0	0.000001
A0A0B4J1Y9	HV372	Immunoglobulin heavy variable 3-72	IGHV3-72	0.001039537	0.035424
Q8IYU8	MICU2	Calcium uptake protein 2, mitochondrial	MICU2	0	0.000001
Q969X1	LFG3	Protein lifeguard 3	TMBIM1	0	0.000001
Q96EY7	PTCD3	Pentatricopeptide repeat domain-containing protein 3	PTCD3	0	0.000001
P02686	MBP	Myelin basic protein	MBP	-	0.000001
O95749	GGPPS	Geranylgeranyl pyrophosphate synthase	GGPS1	0	0.000001
O60701	UGDH	UDP-glucose 6-dehydrogenase	UGDH	0	0.000001
Q96AM1	MRGRF	Mas-related G-protein coupled receptor member F	MRGPRF	0	0.000001
P02008	HBAZ	Hemoglobin subunit zeta	HBZ	0.261721348	0.001579
Q9BYD3	RM04	39S ribosomal protein L4, mitochondrial	MRPL4	0	0.000001
Q8NBN7	RDH13	Retinol dehydrogenase 13	RDH13	0	0.000001
Q9Y2Y8	PRG3	Proteoglycan 3	PRG3	-	0.000001
