# Supplementary material for: Data-independent acquisition-based quantitative proteomic analysis of m.3243A>G MELAS reveals novel potential pathogenesis and therapeutic targets
Source: Medicine (Baltimore). 2022 Oct 14;101(41):e30938. doi: 10.1097/MD.0000000000030938 (PMC9575705; doi:10.1097/MD.0000000000030938)
Supplement: Supplementary file 2 [file medi-101-e30938-s002.pdf]

**Supplementary 2 The protein signature of glycometabolism, mitochondrial structure, oxidative stress and antioxidant defense**

| Protein ID | Protein | Protein descriptions | Genes | Fold Change Ratio | P Value |
|---|---|---|---|---|---|
| P00558 | PGK1 | Phosphoglycerate kinase 1 | PGK1 | 1.010862 | 0.909051 |
| P06733 | ENOA | Alpha-enolase | ENO1 | 1.052366 | 0.67941 |
| P04406 | G3P | Glyceraldehyde-3-phosphate dehydrogenase | GAPDH | 0.947695 | 0.679475 |
| P00338 | LDHA | L-lactate dehydrogenase A chain | LDHA | 0.984829 | 0.945883 |
| P21695 | GPDA | Glycerol-3-phosphate dehydrogenase [NAD(+)] | GPD1 | 1.09059 | 0.560181 |
| P46976 | GLYG | Glycogenin-1 | GYG1 | 1.300340247 | 0.007447 |
| P10809 | CH60 | 60 kDa heat shock protein | HSPD1 | 0.943623 | 0.65031 |
| P08559 | ODPA | Pyruvate dehydrogenase E1 component subunit alpha | PDHA1 | 0.905403 | 0.343616 |
| O75390 | CISY | Citrate synthase | CS | 0.920595 | 0.532728 |
| P40939 | ECHA | Trifunctional enzyme subunit alpha | HADHA | 1.238323 | 0.018426 |
| Q8IYU8 | MICU2 | Calcium uptake protein 2 | MICU2 | 0 | 0.000001 |
| Q96EY7 | PTCD3 | Pentatricopeptide repeat domain-containing protein 3 | PTCD3 | 0 | 0.000001 |
| Q8NE62 | CHDH | Choline dehydrogenase | CHDH | 0.329519729 | 0.028366 |
| P11413 | G6PD | Glucose-6-phosphate 1-dehydrogenase | G6PD | 0.143321113 | 0.031176 |
| P49908 | SEPP1 | Selenoprotein P | SELENOP | 0 | 0.000001 |
| P04792 | HSPB1 | Heat shock protein beta-1 | HSPB1 | 1.489312 | 0.0105384 |
| P02511 | CRYAB | Alpha-crystallin B chain | CRYAB | 1.376931 | 0.0228972 |
| P09601 | HMOX1 | Heme oxygenase 1 | HMOX1 | - | 0.000001 |
| P00441 | SODC | Superoxide dismutase [Cu-Zn] | SOD1 | 1.014766 | 0.940828 |
| P04179 | SODM | Superoxide dismutase [Mn] | SOD2 | 1.24379 | 0.546555 |
| P06702 | S10A9 | Protein S100-A9 | S100A9 | 1.311881 | 0.04811 |

The second line showed the proteins of glycometabolism, the third line showed the proteins of mitochondrial structure, the fourth line showed the proteins of oxidative stress and antioxidant defense.
